# Harnessing Artificial Intelligence to assess the impact of nonpharmaceutical interventions on the second wave of the Coronavirus Disease 2019 pandemic across the world

**DOI:** 10.1038/s41598-021-04731-5

**Published:** 2022-01-18

**Authors:** Sile Tao, Nicola Luigi Bragazzi, Jianhong Wu, Bruce Mellado, Jude Dzevela Kong

**Affiliations:** 1 Quartic.ai, Toronto, ON Canada; 2 Africa-Canada Artificial Intelligence and Data Innovation Consortium, Department of Mathematics and Statistics, York University, Toronto, ON M3J 1P3 Canada; 3 School of Physics, Institute for Collider Particle Physics, University of the Witwatersrand, Johannesburg, South Africa; 4 iThemba LABS, National Research Foundation, Somerset West, South Africa

**Keywords:** Applied mathematics, Scientific data, Statistics, Risk factors, Data mining, Machine learning, Statistical methods

## Abstract

In the present paper, we aimed to determine the influence of various non-pharmaceutical interventions (NPIs) enforced during the first wave of COVID-19 across countries on the spreading rate of COVID-19 during the second wave. For this purpose, we took into account national-level climatic, environmental, clinical, health, economic, pollution, social, and demographic factors. We estimated the growth of the first and second wave across countries by fitting a logistic model to daily-reported case numbers, up to the first and second epidemic peaks. We estimated the basic and effective (second wave) reproduction numbers across countries. Next, we used a random forest algorithm to study the association between the growth rate of the second wave and NPIs as well as pre-existing country-specific characteristics. Lastly, we compared the growth rate of the first and second waves of COVID-19. The top three factors associated with the growth of the second wave were body mass index, the number of days that the government sets restrictions on requiring facial coverings outside the home at all times, and restrictions on gatherings of 10 people or less. Artificial intelligence techniques can help scholars as well as decision and policy-makers estimate the effectiveness of public health policies, and implement “smart” interventions, which are as efficacious as stringent ones.

## Introduction

Since late December 2019, an emerging viral pathogen belonging to the *Coronaviridae* family, termed as “Severe Acute Respiratory Syndrome-related Coronavirus type 2” (SARS-CoV-2), has been isolated as the infectious agent responsible for an outbreak of pneumonia cases of unknown etiology. The initial outbreak has spread from the epicenter of the metropolitan city of Wuhan, province of Hubei, mainland China, to neighboring countries, gradually becoming a global pandemic. SARS-CoV-2 causes a generally asymptomatic or mild, but sometimes severe and even life-threatening respiratory infection, named as “Coronavirus Disease 2019” (COVID-19)^1^. On March 11, 2020, the World Health Organization (WHO) announced that the COVID-19 disease had developed from a “Public Health Emergency of International Concern” (PHEIC) into a pandemic^2^, which is still ongoing and is representing a major public health challenge, due to the highly contagious, quickly spreading nature of the virus^3^. The current scenario is further complicated by the circulation of mutant strains of SARS-CoV-2, known as variants of concern (VoCs)^4^, against which currently licensed and available COVID-19 vaccines appear to be less effective^5^. Moreover, vaccination against COVID-19, despite being safe and efficacious, appears to confer protection that tends to decay after a period of 6 months^6^.

The infectious agent has been overwhelming healthcare settings and facilities worldwide: these are facing a shortage of personnel and medical equipment, which has further increased the strain they are bearing. Vaccines have been licensed and approved only recently, which due to the lack of effective drugs, has resulted in the implementation of non-pharmaceutical interventions (NPIs).

According to the definition of the US “Centers for Disease Control and Prevention” (CDC), NPIs can be conceived as “actions, apart from getting vaccinated and taking medicine, that people and communities can take to help slow the spread of illnesses like pandemic flu”. NPIs include actions implemented at the individual level (like enhanced hygiene practices, wearing of face masks^7^, practicing of social/physical distancing^8^, self-isolation, shelter-in-place/stay-at-home requirements^9^, and self-quarantine). They also include interventions implemented at the community level (such as partial/total lockdown^10^, bans on mass gathering events^11^, school^12^ and workplace closure^13^, internal movements, and international traveling restrictions^14^, among others).

Some groups are particularly vulnerable and prone to SARS-CoV-2, including the frail elderly and those with underlying co-morbidities, who are at higher risk for contracting the virus and developing complications. This has suggested the implementation of ad hoc smart^15^ or local^16^ lockdown/quarantine^17^, known also as targeted^18^/precision shielding^19^ measures.

Based on their stringency, NPIs can be classified into eradication *versus* mitigation strategies^20^.

Stringent and drastic measures like nation-wide/global lockdowns have been effective to contain the COVID-19 spreading but are economically and socially unsustainable and highly disruptive^21^.

For this reason, countries and public health authorities have been striving to find the best trade-off possible between COVID-19 induced strictures and relaxing/lifting of NPIs where and when data and epidemiological trends allow to do so. This^22^, together with other factors, such as seasonality, or meteorological/climatic parameters, has resulted in a series of relapses/waves^23^. Due to the emerging nature of the pathogen, with the population being immunologically naïve to SARS-CoV-2, and given the enforcement of NPIs, a significantly large proportion of the population has been kept susceptible during the first wave of COVID-19. Until the achievement of herd immunity, due to the cyclical relaxing and reinstatement of NPIs, several waves of COVID-19 have occurred and further ones are expected to occur until the disease extinction or its transition to endemicity^22^.

### Aim of the study

Given the variety of NPIs that can be implemented and the different possibilities of integrating/incorporating them into packages of public health measures, it is of crucial importance to track and monitor their effectiveness using real-world data generated by public health policies at the global level^24^. Artificial intelligence (AI) and big data can help in this^25^, assisting public health decision- and policy-makers in the complex decision-making process concerning the optimal implementation, enforcement, and timing of lifting and reinstatement of the most effective NPIs.

In the present paper, we will explore the impact of NPIs on the second wave of COVID-19 utilizing AI techniques, taking into account pre-existing country-specific characteristics (for example, economic-financial, socio-demographic, and environmental parameters).

## Materials and methods

All codes are available on a Github repository https://github.com/sit836/covid. For the main objective of this paper, the dependent variable is the effective reproduction number and the covariates are the NPIs and climatic, environmental, clinical, health, economic, pollution, social, and demographic (CECHEPSD) variables that can explain the epidemiological trends of the COVID-19 pandemic (Tables S1–S5).

### Spreading rates of COVID-19 during the first and second waves across the globe

To get the values of the spreading rates of COVID-19 during the first (*r*_1_) and second (*r*_2_) waves for each country, we use Python’s SciPy curve fit function to fit the rate of change in cumulative cases of a logistic growth model to daily confirmed cases^26^. A statistical model was used because a mechanistic model would require a complex parameterization procedure. This would be characterized by a high degree of uncertainty, especially during the early phases of an outbreak, due to the lack of detailed data. Statistical models are data-driven, and thus do not suffer from such shortcomings. Among the statistical models, we were encouraged by the work of Ref.^27^ to choose a logistic model. Ma et al.^27^ compared four commonly used statistical models (namely, exponential, Richards, logistic, and delayed logistic models), and found out that the logistic model outperforms the others in estimating the growth of epidemics. Moreover, the logistic models have been extensively utilized to provide reliable estimations of the upper and lower bounds of COVID 19 related scenarios^28,29^. In the logistic model, the cumulative number of cases *c*(*t*) satisfies:

1 $$\text{c}\left(\text{t}\right)=\frac{\text{K}}{\left(1+\left[\frac{\text{K}}{\text{c}\left(0\right)}-1\right]{\text{e}}^{-\text{rt}}\right)},$$

where *K* is the total number of people infected at the end of the outbreak, *r* the speed of the epidemic growth, and c(0) the initial number of cases. The change in cumulative cases that is fitted to the 7-day rolling mean of daily confirmed cases is given as *I*(*t*) = *c*(*t* + 1) − *c*(*t*), where t is a small increment in time (taken to be a day). We fit the change in the cumulative cases rather than the cumulative cases, because observations drawn from the same cumulative curve are correlated. Most curve fitting algorithms assume that the errors in individual observations are statistically independent; this is not true with cumulative data where each observation contains all of the cases from previous observations. For this reason, to avoid such assumptions and shortcomings, we utilized the least square fitting algorithm. We truncated all COVID-19 reported daily case time series within the window of the first and second waves, to the day with the highest daily count, because some countries have lingered near peak daily count for much longer than a logistic growth model would predict, which would pull the model peak to later than the actual date of peak incidence and thereby underestimate the spreading rate. We manually checked each time series and ensured that the highest daily count only occurred during a peak. We only consider countries that experienced at least two waves. Also, we include only countries that were at least 6 days into a period (for both the first and second wave periods) with at least 30 daily cases as of July 29, 2020, after truncating at the peak. The time when countries observed their first 30 daily case count was considered the initial time. The first wave is based on a fitting window from the initial time until peak time and that for the second wave is based on a fitting window from the time at which a country records the lowest number of daily cases between the first and second peak to the second peak.

We eliminated countries whose logistic growth model has *R*^2^ less than 0*.*95 for any of the fits (first and second waves). This is to ensure that we only include countries that our model can explain at least 95% of the variations in their spreading rates.

Some countries do not report COVID-19 cases on a daily basis; some countries have variable reporting delays, and some may have changed reporting methods resulting in dramatic spikes in cases for particular dates. To circumvent this inaccuracy in date, we used the 7-day rolling average (right-aligned) for daily cases.

*R*_*e*_ can serve as a baseline expectation for estimating how fast COVID-19 would spread if all interventions were prematurely lifted prior to the start of the second wave, given that the percentage of the population susceptible to COVID-19 was still relatively high at that time.

Using these growth rates, we calculated the basic reproduction number of COVID 19 (*R*_0_) (first wave) and the effective reproduction number of the second wave (*R*_*e*_) as follows^30^:

$${R}_{0}={e}^{{r}_{1}T}, {R}_{e}={e}^{{r}_{2}T},$$

where *T* is the serial interval of COVID-19 (time delay between the symptom onset of a primary case and the secondary cases). The value *T* for COVID-19 lies in the interval^4,8,31–33^.

### Covariates

Next, we compiled data on the covariates.

#### Non-pharmaceutical interventions (NPIs)

We compiled data on 18 common policy responses that governments across the globe have taken to respond to the pandemic. These include school and workplace closures, cancellation of public events and gatherings, stay-at-home orders, and international and domestic travel restrictions: these have been extracted from https://github.com/OxCGRT/covid-policytracker/blob/master/documentation/codebook.md. Each NPI is an indicator recorded on an ordinal scale where the larger the index, the stricter the policy (Tables S2, S3). The dataset records governmental responses implemented in the year 2020 for several countries.

#### CECHEPSD variables

Data on several climatic, environmental, clinical, health, economic, pollution, social, and demographic variables were obtained from publicly available databases (see Table S4 for the full list of variables and references).

### Pre-processing data

We kept only countries having both first and second waves. Then we filtered out covariates if the missing ratio is greater than 10% and replaced the missing values (2% of data) with the mode. Next, we removed variables, such as country name and cumulative cases per million population, whose value either does not add any information to the model or would not actually be available at the time we want to make a prediction. In the end, we converted categorical variables into integers.

We represented temporal policy responses as time-independent numerical values. First, for every country and policy, we ignored cells with no measures and counted the number of days lasting for each possible action. For example, regarding the policy of canceling public events, assume Canada recommended canceling public events for 30 days and required canceling for 60 days. Then we used the tuple (30, 60) to represent the information of such a policy. Next, we imputed the missing values (0.03% of data) with the mode.

In the end, we have 55 countries and 35 covariates when we studied the association between *R*_0_ and CECHEPSD variables; 53 countries and 73 covariates when we regressed ${\widehat{R}}_{e}-{R}_{e}$ on NPIs; 53 countries and 108 covariates when we regressed the growth rate of the second wave on NPIs and CECHEPSD variables.

#### Evaluation metrics

We adopted the mean squared error (MSE) and the coefficient of determination *R*^2^ as the evaluation measures. MSE measures the average of the squares of the errors.

$$MSE=\frac{1}{n}\sum_{i=1}^{n}{\left({y}_{i}-{\widehat{y}}_{i}\right)}^{2},$$

where *n* is the sample size, ${y}_{i}$ is the observed value and ${\widehat{y}}_{i}$ is the predicted one; *R*^2^ measures how well a model performs compared to naïve average forecasting

$${R}^{2}=1-\frac{\sum_{i=1}^{n}{\left({y}_{i}-{\widehat{y}}_{i}\right)}^{2}}{\sum_{i=1}^{n}{\left({y}_{i}-\overline{{y }_{i}}\right)}^{2}},$$

where $\overline{{y }_{i}}$ is the average of the observed values. It is worth pointing out that *R*^2^ in our definition can be negative if the model predictions are far away from the actual values.

### Random forest regression analysis of the association between CECHEPSD, NPIs and *R*_*e*_

We used random forests (RF), an “off-the-shelf” machine learning algorithm, to predict *R*_*e*_ based on 18 CECHEPSD, and 8 NPIs variables. Suppose the inputs pairs are (*x*_1_*, y*_1_),*…,*(*x*_*n*_, *y*_*n*_), where *x*_*i*_* ∈ *R^*p*^ and *y*_*i*_* ∈ *R*.* Every decision tree in a forest forms a step function over a partition *R*_1_, *R*_2_,*…*, *R*_*M*_:

$${\text{f}}\left( {\text{x}} \right) = \sum\limits_{{{\text{m}} = 1}}^{{\text{M}}} {{\text{c}}_{{\text{m}}} } {\text{I}}_{{{\text{R}}_{{\text{m}}} }} \left( {\text{x}} \right),$$

where *c*_*m*_ are model parameters and *I*_*Rm*_ is an indicator function:

$${\text{I}}_{{{\text{R}}_{{\text{m}}} }} \left( {\text{x}} \right) = \left\{ {\begin{array}{*{20}c} {1,} & {x \in {\text{R}}_{{\text{m}}} } \\ {0,} & {otherwise} \\ \end{array} } \right.$$

RF builds a large collection of de-correlated trees and then averages them. Differently from generalized additive models (GAMs), RF is a decision tree-based method, which can capture the interactions among covariates. Therefore, in practice, we expect RF to almost always outperform GAM. In the implementation, we used the “RandomForestRegressor” module in the Python Scikit-learn library.

For regression, we grew and combined 500 decision trees. Each tree is grown with the randomly selected square root of the total number of covariates when making splits^34^ and it has the maximum depth found via tenfold cross-validation. A large number of trees was used to stabilize feature importance measures. If the number of trees is not large enough, then some covariates will not be given a chance to play a role in each tree. For low complexity trees, increasing the number of trees will not cause over-fitting^35^.

To understand how covariates are contributing to the model fitting, we used Breiman’s^35^ permutation-based measures, which assess the importance of a feature by calculating the increase in the model’s error after permuting the feature. A feature is important if shuffling its values increases the model error, because, in this case, the model relied on the feature. A feature is unimportant if shuffling its values does not change the model’s error much.

### Comparing the growth rate of the first wave and second waves

Here we compare the spreading rates of the first wave and second waves of COVID-19 across countries. To account for susceptible population, we instead compared the ${R}_{e}$ and ${\widehat{R}}_{e}$, which is the product of the predicted value *R*_0_ and the fraction of susceptible population at the start of the second wave.

We would like to know whether the centers of the observations ${\text{R}}_{\text{e}}$’s and ${\widehat{\text{R}}}_{\text{e}}$’s are statistically different. If there is no statistical difference between the centers of ${\widehat{\text{R}}}_{\text{e}}$ and R_e_, it is likely that partial lifting of NPIs led to the spreading rate of the second wave of COVID-19 to be statistically similar to what it was during the first wave (after accounting for those with partial immunity). One explanation for this could be that the NPIs that had the greatest impact on the spreading rate were lifted.

We investigated the shape of distributions for ${R}_{e}$ and ${\widehat{R}}_{e}$ via Kolmogorov–Smirnov tests. Both P-values are extremely small: One for ${R}_{e}$ is 5*.*7 × 10^−47^ and the other _for_ ${\widehat{R}}_{e}$ is 3*.*1 × 10^−54^. Also, the side-by-side boxplots of ${R}_{e}$ and ${\widehat{R}}_{e}$ (Fig. S3) show that data in either group are not symmetric. Therefore, we decided to use Wilcoxon signed-rank test, a non-parametric analogy to the classical paired *t* test to test whether the two populations have the same distribution. If the null hypothesis is rejected, then we have evidence that the centers of the two populations differ.

### Ethics and consent

All authors have been personally and actively involved in substantial work leading to the paper, and will take public responsibility for its content.

## Results

### Estimation of the spreading rates of COVID-19 during the first and second waves

**Figure 1:**
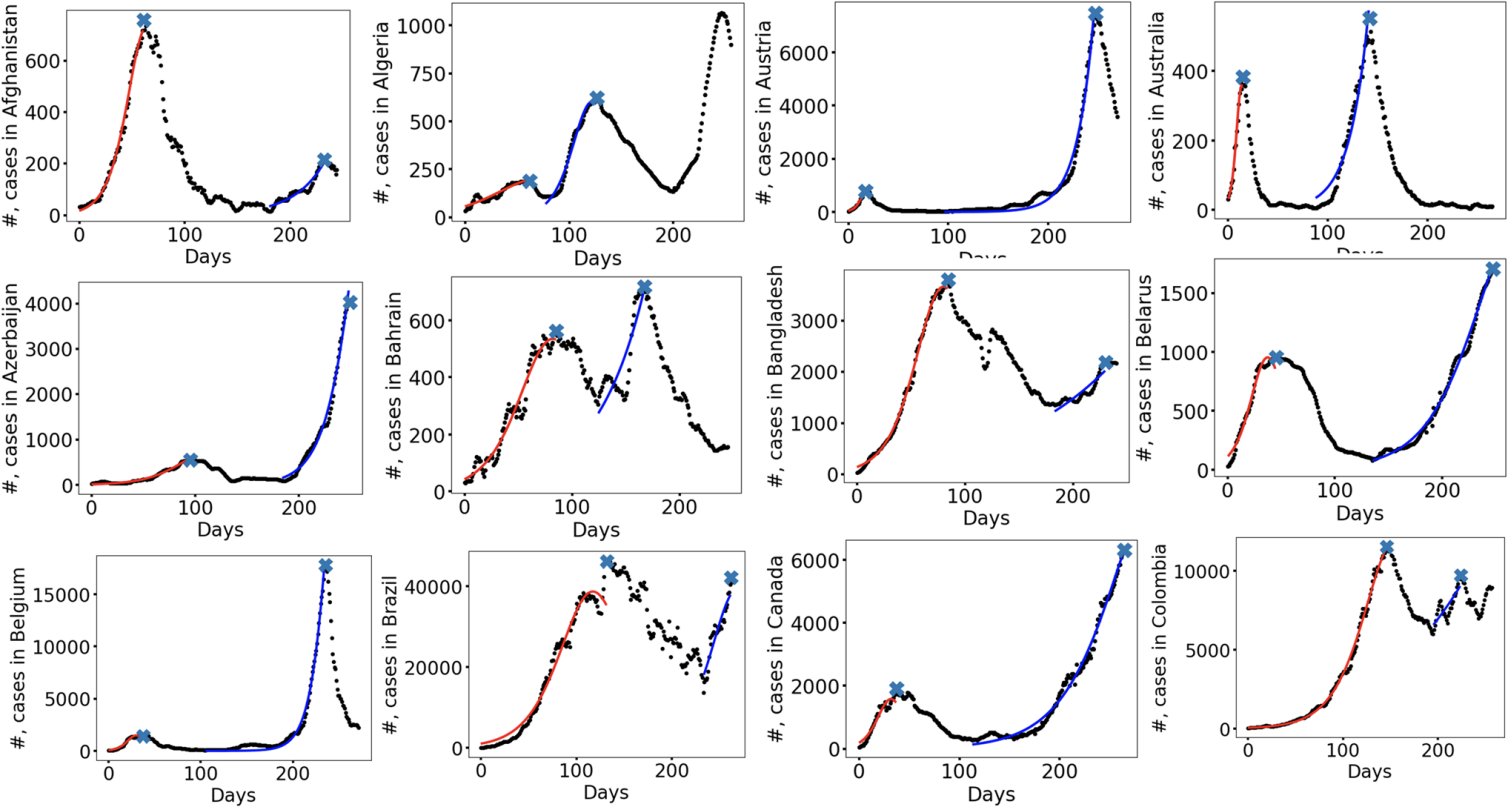
The time course dynamics of COVID-19. The red line corresponds to the first COVID-19 wave, the blue line to the second COVID-19 wave, the blue cross to the peak, and the dotted line to real data.

Figure 1, Figs. S1 and S2 show the growth curves fitted to the observed time-series of daily confirmed cases across countries. The plotted estimate for the first wave is based on a fitting window from the initial time until peak time and that for the second wave is based on a fitting window from the time between the first and the second peak with the lowest number of cases until the second peak time. Only countries whose logistic growth model had an *R*^2^ of or greater than 0.95 were considered.

**Figure 2:**
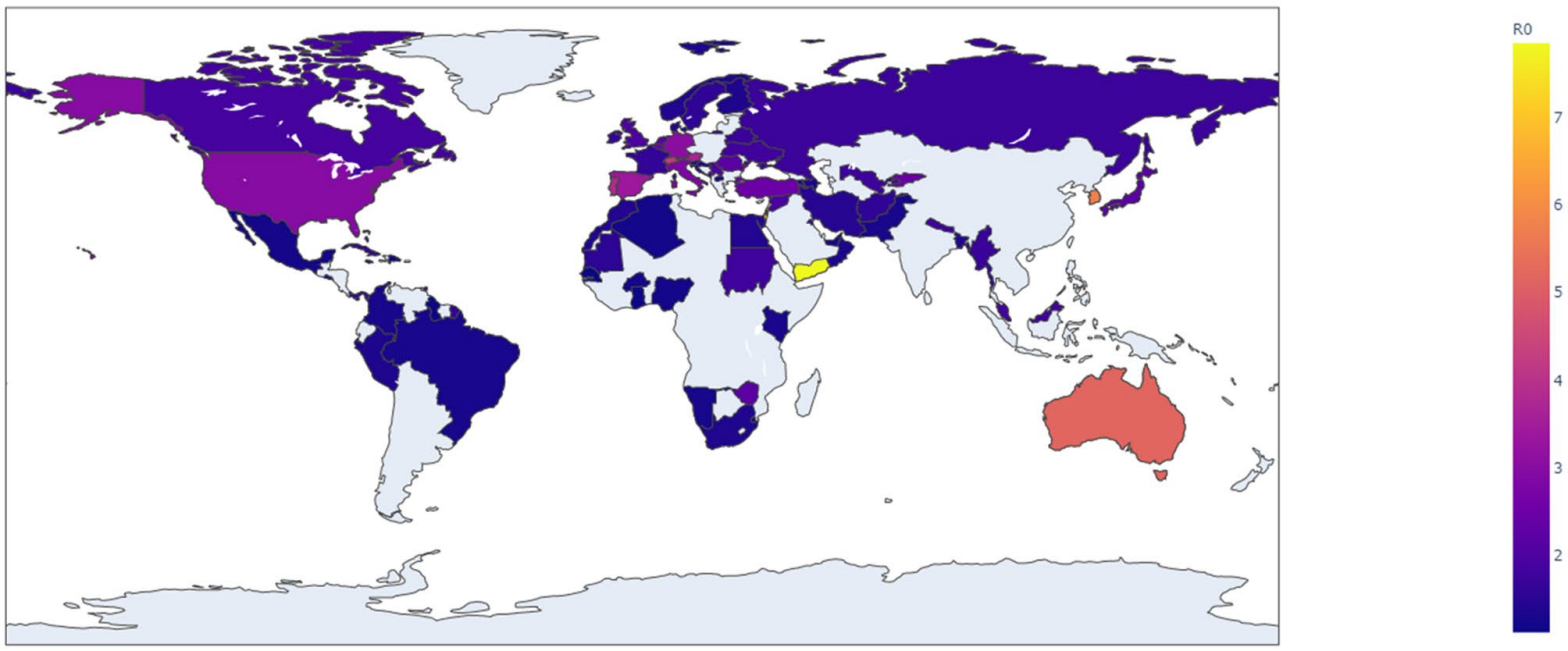
The basic reproduction number of COVID-19 across countries.

**Figure 3:**
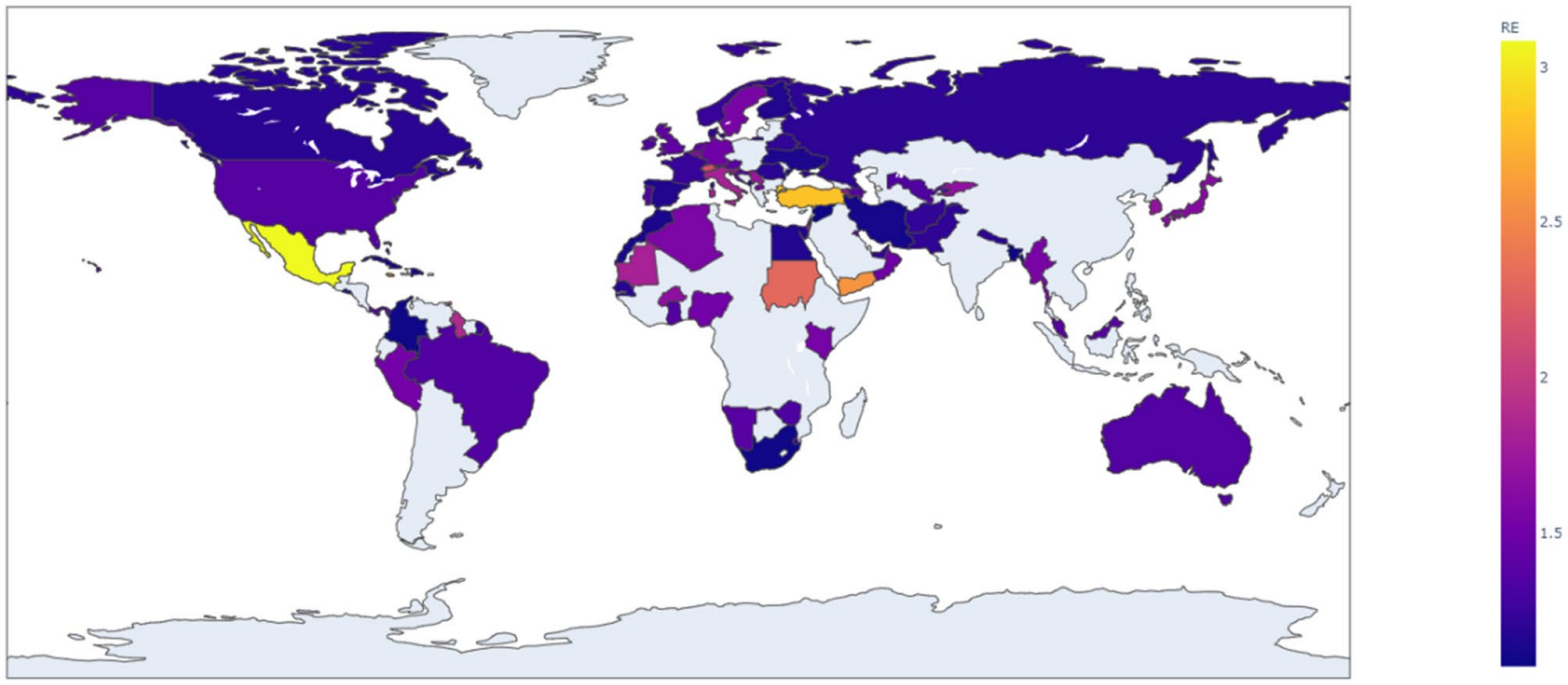
The effective reproduction number of COVID-19 across countries during the second wave.

Figure 2 and the second column in Table S5 summarize the estimated basic reproduction number *R*_0_ across countries while Fig. 3 and the third column in Table S5 summarize the estimated effective reproduction number (second wave) *R*_*e*_ across countries. Figures 2 and 3 were created using Ploty.py 4.14.3, a Python open source library (https://plotly.com/graphing-libraries/).

*R*_0_ and *R*_*e*_ are highest in Israel (*R*_0_ = 6*.*93) and Mexico (*R*_*e*_ = 3*.*08) respectively. The lowest *R*_0_ and *R*_*e*_ were respectively estimated in Senegal (*R*_0_ = 1*.*13) and Bangladesh (*R*_*e*_ = 1*.*07). Overall, the mean *R*_0_ and *R*_*e*_ were respectively 2*.*02 (*S.D.* 1*.*09) and 1*.*45 (*S.D.* 0*.*41). The United Kingdom (*R*_0_ = 2*.*01), Luxembourg (*R*_0_ = 1*.*90) and the Netherlands (*R*_0_ = 2*.*17) had *R*_0_ values that were closer to the mean *R*_0_ value, while the Netherlands (*R*_*e*_ = 1*.*45), Oman (*R*_*e*_ = 1*.*50), and Namibia (*R*_*e*_ = 1*.*39) had *R*_*e*_ values closer to the mean *R*_*e*_. We used the mean and standard deviation as descriptive statistics for R_e_ and R_0_ because we observed that they are normally distributed across countries.

### Association between NPIs, CECHEPSD variables and growth rate of the second wave

Growing a RF with 500 trees and maximum depth = 2 gives MSE 0.08 and *R*^2^ 0.51. We compared RF with the least absolute shrinkage and selection operator (LASSO) regression^36^ with regularization parameter = 0.3. The value of the regularization parameter was found via tenfold cross-validation on the normalized covariates. LASSO gives MSE 0.16 and *R*^2^ 0.00 which are of several orders of magnitude worse than RF, since LASSO does not take nonlinearity into account.

**Figure 4:**
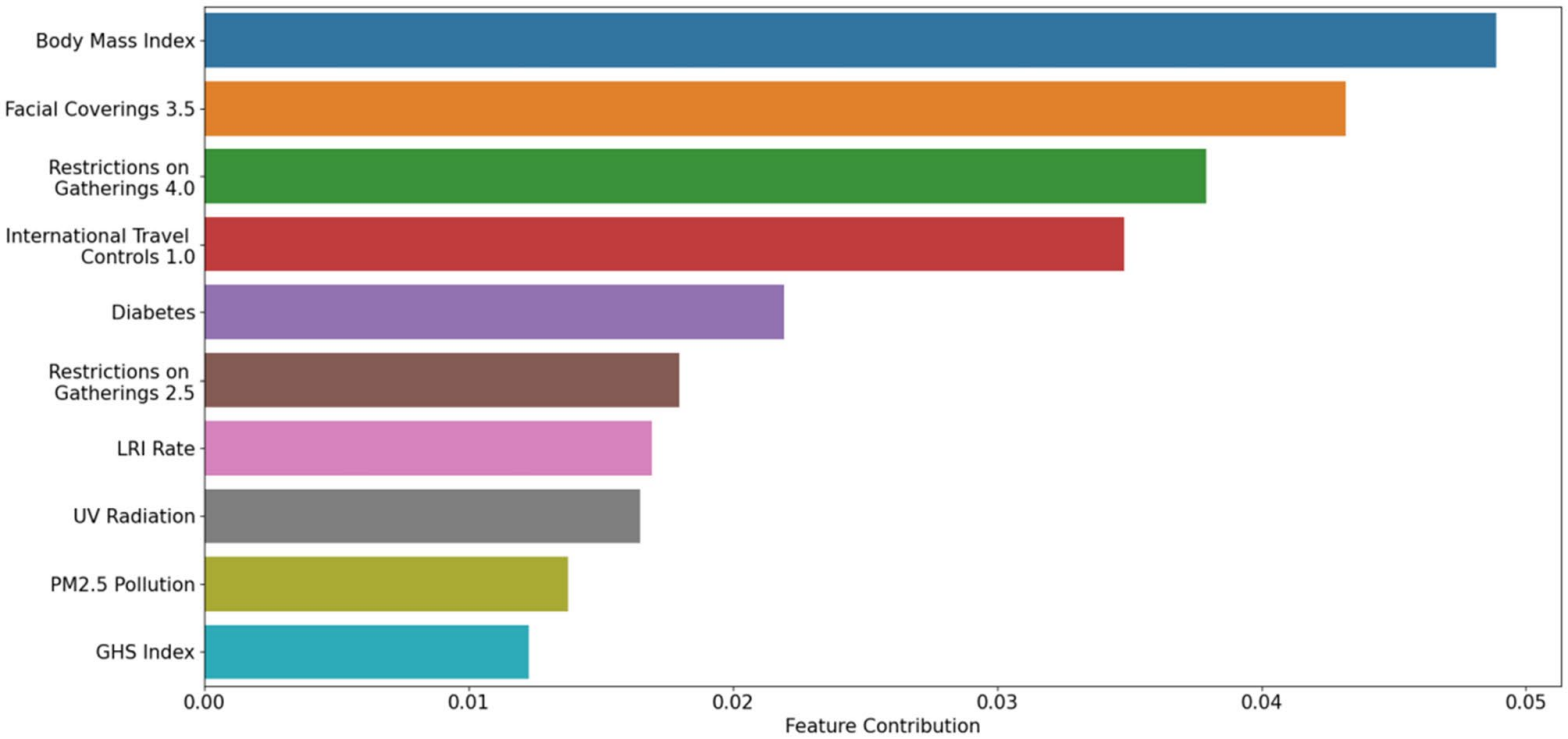
Features importance (Top 10): (i) body mass index, (ii) the number of days that the government sets restrictions on requiring facial coverings outside the home at all times, (iii) restrictions on gatherings 4.0, (iv) screened foreign travelers on international travel, (v) diabetes prevalence, (vi) restrictions on gatherings 2.5, (vii) LRI rate, (viii) UV radiation, (ix) PM2.5 air pollution, and (x) GHS index.

Figure 4 indicates that average body mass index (BMI) was the first most important variable associated with the growth rate of the second wave. The second variable in terms of importance is the number of days that the government sets restrictions on requiring facial coverings outside the home at all times regardless of location or presence of other people in some areas. Restrictions on gatherings of 10 people or less, and screened foreign travelers on international travel are the third and fourth most important variables associated with the growth rate of the second wave, respectively.

### Hypothesis testing for difference of medians between of ${{\varvec{R}}}_{{\varvec{e}}}$ and ${\widehat{{\varvec{R}}}}_{{\varvec{e}}}$

The value of the statistical Wilcoxon test is 169.0 and P-value is 4*.*8 × 10^−7^. The null hypothesis is rejected. It suggests that the actual observations and the estimates are unlikely from the same distribution. Therefore, a statistically significant difference exists between the two medians. Thus, it is likely that the partial lifting of NPIs did not cause the spreading rate of the second wave of COVID-19 to be statistically similar to what it was during the first wave (after accounting for those with partial immunity).

## Discussion

In the present investigation, we found that (i) body mass index, (ii) the number of days that the government sets restrictions on requiring facial coverings outside the home at all times regardless of location or presence of other people in some areas, and (iii) restrictions on gatherings of 10 people or less are the three most important variables in the model. Among health-related variables, body mass index has been found to be associated with COVID-19. Sarmadi et al.^37^ have performed an ecological study, utilizing global databases (from the WHO and the NCD Risk Factor Collaboration, or NCD-RisC), to dissect the correlation between age-standardized body mass index and the risk of contracting COVID-19 in terms of incidence and mortality ratio. Authors were able to find a positive correlation, which was stronger in nations and territories with younger populations (like developing countries). Such a correlation remained statistically significant after adjusting for confounding factors (such as socio-demographic and economic parameters). This finding has epidemiological relevance, in that it has practical implications in terms of public health policies. Health decision- and policy-makers could devise and implement interventions aimed at monitoring and counteracting overweight and obesity, promoting health literacy and the adoption of healthy lifestyles, mitigating, in this way, the burden of disease imposed by high body mass index. The other two of the three most significant variables are NPIs. Quantifying the efficacy of mitigation strategies against an outbreak caused by an emerging pathogen is of paramount importance^38^, both to avoid further waves/relapses of the same outbreak and to guide future preparedness response plans^39^.

Despite the importance of tracking and monitoring the effectiveness of NPIs, there are few large-scale studies conducted at the global level. Exploring this topic is technically challenging because the variables under study are highly intercorrelated, exhibiting spatial, temporal, and spatio-temporal clustering patterns^40^. There exist, instead, several studies estimating the impact of single individual NPIs at the country-level or in a group of countries, whereas a comprehensive assessment of all the NPIs being implemented (enforced/lifted) is necessary. James and Menzies^41^ have investigated changes in numerous aspects of COVID-19 related behaviors between the first and second waves, for example in terms of outbreak severity across the United States, where each state has individually responded to the pandemic. Authors have developed a formal definition and mathematical framework to properly classify COVID-19 surges/peaks, differentiating between a first and second wave, and have compared the various infectious trajectories across states to identify the most effective pandemic responses. In a second paper, James et al.^42^ have extended their analytical techniques to incorporate European countries as well, demonstrating substantial heterogeneity within Europe and the United States. In a subsequent paper, James et al.^43^ have compared three countries most hardly hit by the outbreak, namely the United States, India, and Brazil, assessing patterns of similarity and dissimilarity in the response to the pandemic.

In a previous study^44^, we analyzed the effects of the implementation of NPIs on the initial growth rate of COVID-19, taking into account as well CECHEPSD variables, using a multiple linear regression model and incorporating 29 parameters. Out of these 29 variables, ten (8 CECHEPSD characteristics and 2 NPIs) were found to correlate with the initial growth of COVID-19. In particular, the population residing in urban agglomerations (centers of more than 1 million inhabitants), atmospheric fine particulate matter (PM2.5) air pollution mean annual exposure, life expectancy, number of hospital/healthcare setting beds available, urban population, Global Health Security (GHS) index, and international movement restrictions were the parameters which had the most significant impact on the initial growth of COVID-19. Based on these findings, we concluded that, among NPIs being implemented, only one (namely, restrictions on international movements) was found to have a relative significance with respect to the initial growth rate of COVID-19, whilst CECHEPSD factors seemed to play a more prominent role in the initial growth rate of COVID-19 and its transmission dynamics.

A study^39^ attempted to quantitatively assess the effects of NPIs enforced in several countries/territories in terms of changes in the COVID-19 effective reproduction number, employing an integrative modeling approach, combining classical inference, bio-statistics, and AI techniques. The authors utilized a training dataset of 6068 hierarchically coded NPIs from 79 countries and a validated external database merging two datasets, including 42,151 additional NPIs from 226 countries. Authors were able to find that a highly disruptive, costly, intrusive NPI like a national lockdown was as effective as a package of less drastic and stringent NPIs. In particular, the largest effects in terms of reduction in the effective reproduction number were found for NPIs like the ban of small gathering events, school closure, and border control/restrictions.

Liu et al.^40^ obtained similar findings, utilizing hierarchical clustering and panel, longitudinal regression tools to quantify the efficacy of 13 NPI-related categories in the study period January–June 2020. The authors found that two NPIs (closure of educational institutions and internal movement restrictions) were particularly efficacious in decreasing time-varying reproduction numbers. Other NPIs (namely, workplace closure, debt/contract relief, income support, cancellation of public events, and gathering events ban/restrictions) were effective as well. Evidence concerning other mitigation strategies (such as shelter-in-place/stay-at-home orders, public information awareness campaigns, public transportation closure, travel restrictions, testing, and contact tracing) was, instead, contrasting.

Li et al.^45^ conducted a modeling study on the effect of escalating/de-escalating NPIs in terms of variation of the COVID-19 reproduction number in the period January–July 20, 2020, collecting data from 131 countries. Authors found that NPIs like school closure, workplace closure, public events cancellation/ban, shelter-in-place/stay-at-home orders, and internal movement restrictions were able to curtail the spreading of the virus, with bans on public events achieving the statistical significance threshold. Lifting of bans on public gathering events and reopening of schools resulted in a significant increase in the COVID-19 reproduction number.

Bo et al.^46^ analyzed 1,908,197 confirmed COVID-19 cases from 190 countries in the period January–April 2020, categorizing NPIs as mandatory face-mask use in public, self-isolation/quarantine, social/physical distancing, and traffic controls/restrictions. These resulted in a decrease in the COVID-19 reproduction number, which was more marked when a coherent, integrated package of public health interventions was implemented and enforced.

Our investigation confirmed the usefulness of NPIs implemented worldwide, complementing and adding to the existing literature. The strength of the present paper is, indeed, the fact that we used a quite large list of covariates and NPIs to discern their association with the growth rate of the second wave of COVID-19. However, this list is far from being exhaustive and other covariates could have been included, given that the literature on the determinants of COVID-19 is constantly under flux and continuously evolving. For example, there is evidence of causal correlations between COVID-19 and PM10^47^ as well as between COVID-19 and relative humidity^48^. Furthermore, the PM2.5 characteristic analyzed in this paper is the mean annual exposure, while some papers have found correlations with the exceeding of daily thresholds^49^. This warrants further research exploring other covariates for which recent studies have shown causal associations.

In conclusion, extremely aggressive measures like nation-wide lockdowns, have significantly contributed to the containment of the COVID-19 pandemic, by curbing the SARS-CoV-2 transmission dynamics, and saving lives, but, on the other hand, have imposed a dramatically high societal and economic burden. Advanced data mining techniques, including approaches relying on Big Data and AI, can enable scholars as well as public health decision- and policy-makers to estimate the effectiveness of public health policies and mitigation strategies to counteract the toll of the outbreak in terms of infections and deaths, enforcing and implementing “smart” interventions, which are as efficacious as drastic and stringent ones.

## Supplementary Information

Supplementary Information.
