# Supplementary material for: Harnessing Artificial Intelligence to assess the impact of nonpharmaceutical interventions on the second wave of the Coronavirus Disease 2019 pandemic across the world
Source: Sci Rep. 2022 Jan 18;12:944. doi: 10.1038/s41598-021-04731-5 (PMC8766477; doi:10.1038/s41598-021-04731-5)
Supplement: Supplementary file 1 — Supplementary Information. [file 41598_2021_4731_MOESM1_ESM.pdf]

Harnessing Artificial Intelligence to assess the impact of nonpharmaceutical interventions on the second wave of the Coronavirus Disease 2019 pandemic across the world

Sile Tao[1,*], Nicola Luigi Bragazzi[2,*], Jianhong Wu[2], Bruce Mellado[3,4], and Jude Dzevela Kong[2,†]

[1]Quartic.ai, Toronto, Ontario, Canada.
[2]Africa-Canada Artificial Intelligence and Data Innovation Consortium, Department of Mathematics and Statistics, York University, Toronto, ON M3J 1P3, Canada.
[3]Department of Zoology, University of British Columbia, BC V6T 1Z4, Canada.
[3]School of Physics, Institute for Collider Particle Physics, University of the Witwatersrand, Johannesburg, South Africa.
[4]iThemba LABS, National Research Foundation, Somerset West, South Africa.
[*]Co-first authors
[†]Corresponding author's email: jdkong@yorku.ca

December 14, 2021

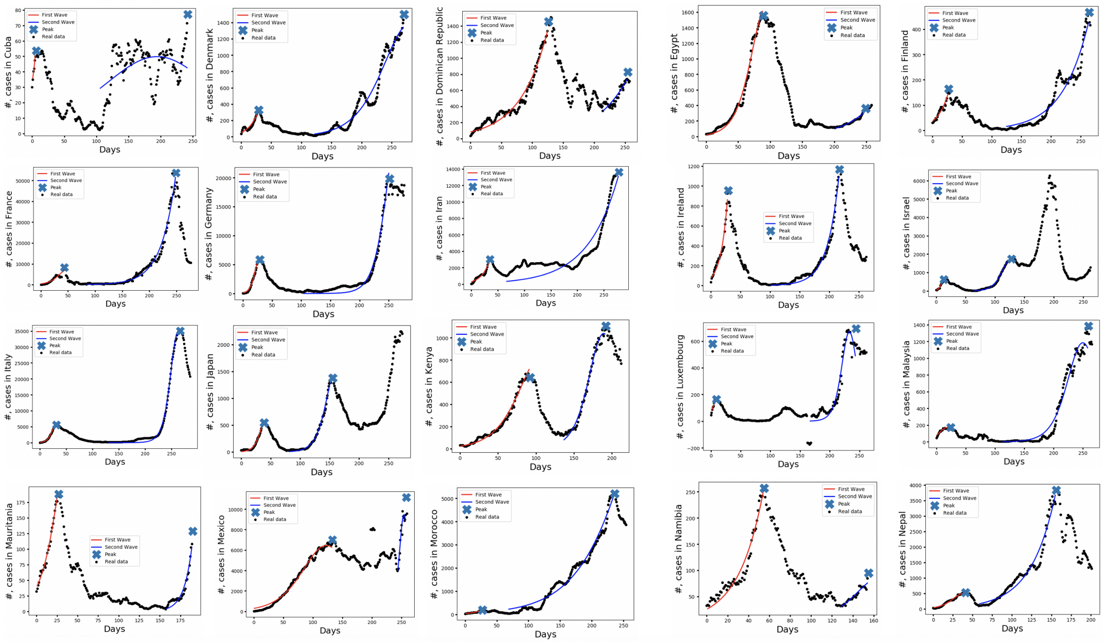

Figure 1: The time course dynamics of COVID-19

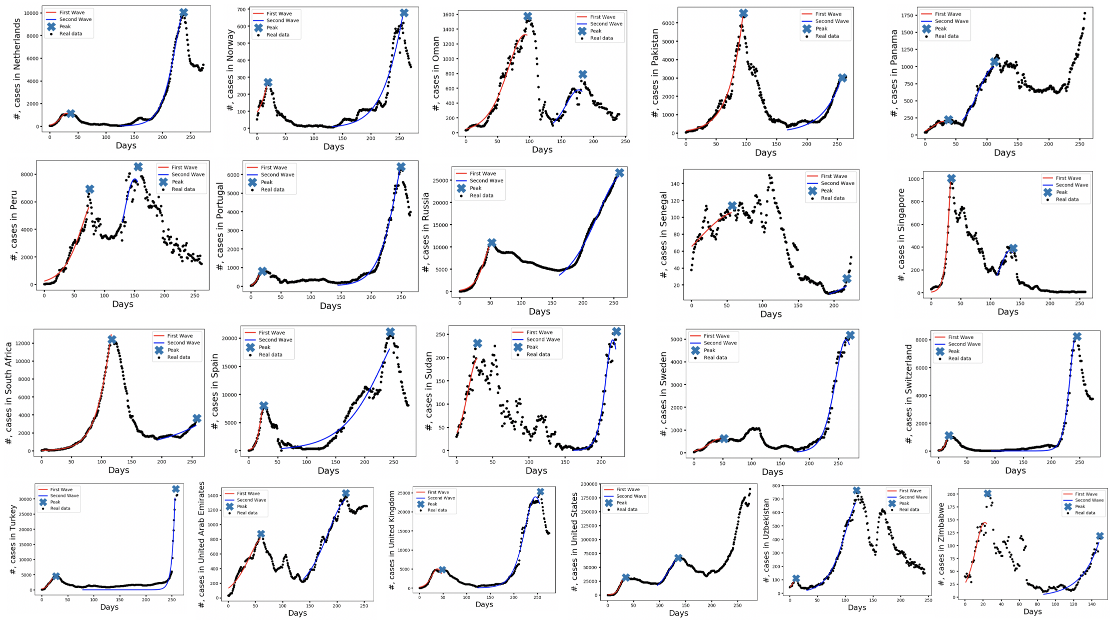

Figure 2: The time course dynamics of COVID-19

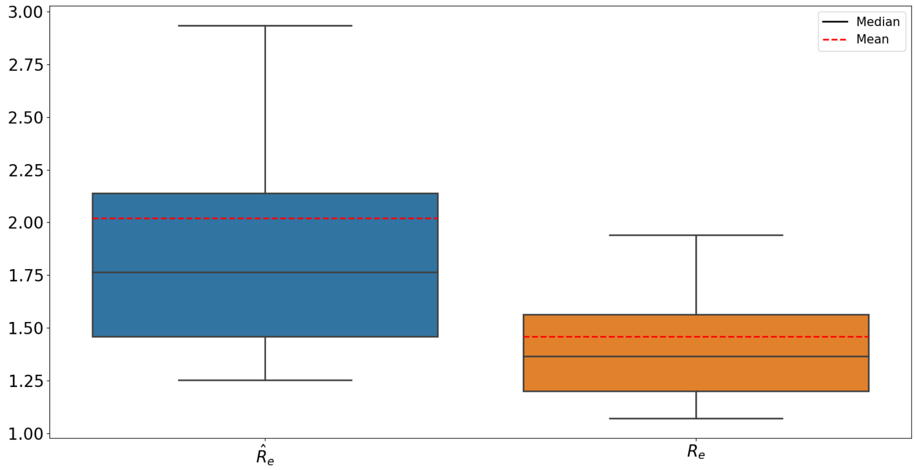

Figure 3: Distribution of growth rate of the first wave and second waves

Table 1: Containment and closure policies

Name	Description	Coding
School closing	Record closings of schools and universities	0 - no measures 1 - recommend closing or all schools open with alterations resulting in significant differences compared to non-Covid-19 operations 2 - require closing (only some levels or categories, e.g., just high school, or just public schools) 3 - require closing all levels Blank - no data

Workplace closing	Record closings of workplaces	0 - no measures 1 - recommend closing (or recommend work from home) 2 - require closing (or work from home) for some sectors or categories of workers 3 - require closing (or work from home) for all-but-essential workplaces (e.g., grocery stores, doctors) Blank - no data
Cancel public events	Record cancelling public events	0 - no restrictions 1 - restrictions on very large gatherings (the limit is above 1,000 people) 2 - restrictions on gatherings between 101-1,000 people 3 - restrictions on gatherings between 11-100 people 4 - restrictions on gatherings of 10 people or less Blank - no data
Restrictions on gatherings	Record limits on gatherings	0 - no restrictions 1 - restrictions on very large gatherings (the limit is above 1,000 people) 2 - restrictions on gatherings between 101-1,000 people 3 - restrictions on gatherings between 11-100 people 4 - restrictions on gatherings of 10 people or less Blank - no data

Close public transport	Record closing of public transport	0 - no measures 1 - recommend closing (or significantly reduce volume/route/means of transport available) 2 - require closing (or prohibit most citizens from using it) Blank - no data
Stay at home requirements	Record orders to "shelter-in-place" and otherwise confine to the home	0 - no measures 1 - recommend not leaving house 2 - require not leaving house with exceptions for daily exercise, grocery shopping, and 'essential' trips 3 - require not leaving house with minimal exceptions (e.g., allowed to leave once a week, or only one person can leave at a time, etc) Blank - no data
Restrictions on internal movement	Record restrictions on internal movement between cities or regions	0 - no measures 1 - recommend not to travel between regions/cities 2 - internal movement restrictions in place Blank - no data
International travel controls	Record restrictions on international travel. Note: this records policy for foreign travellers, not citizens	0 - no restrictions 1 - screening arrivals 2 - quarantine arrivals from some or all regions 3 - ban arrivals from some regions 4 - ban on all regions or total border closure Blank - no data

Table 2: Economic policies

Name	Description	Coding
Income support	Record if the government is providing direct cash payments to people who lose their jobs or cannot work. Note: only includes payments to firms if explicitly linked to payroll/salaries	0 - no income support 1 - government is replacing less than 50% of lost salary (or if a flat sum, it is less than 50% median salary) 2 - government is replacing 50% or more of lost salary (or if a flat sum, it is greater than 50% median salary) Blank - no data
Debt/contract relief	Record if the government is freezing financial obligations for households (eg stopping loan repayments, preventing services like water from stopping, or banning evictions)	0 - no debt/contract relief 1 - narrow relief, specific to one kind of contract 2 - broad debt or contract relief
Fiscal measures	Announced economic stimulus spending. Note: only record amount additional to previously announced spending	Record monetary value in USD of fiscal stimuli, includes any spending or tax cuts NOT included in E4, H4 or H5. 0 - no new spending that day Blank - no data
International support	Announced offers of Covid-19 related aid spending to other countries. Note: only record amount additional to previously announced spending	Record monetary value in USD 0 - no new spending that day Blank - no data

Table 3: Health system policies

Name	Description	Coding

Public information campaigns	Record presence of public info campaigns	0 - no Covid-19 public information campaign 1 - public officials urging caution about Covid-19 2- coordinated public information campaign (e.g., across traditional and social media) Blank - no data
Testing policy	Record government policy on who has access to testing. Note: this records policies about testing for current infection (PCR tests) not testing for immunity (antibody test)	0 - no testing policy 1 - only those who both (a) have symptoms AND (b) meet specific criteria (eg key workers, admitted to hospital, came into contact with a known case, returned from overseas) 2 - testing of anyone showing Covid-19 symptoms 3 - open public testing (eg "drive through" testing available to asymptomatic people) Blank - no data
Contact tracing	Record government policy on contact tracing after a positive diagnosis. Note: we are looking for policies that would identify all people potentially exposed to Covid-19; voluntary bluetooth apps are unlikely to achieve this	0 - no contact tracing 1 - limited contact tracing; not done for all cases 2 - comprehensive contact tracing; done for all identified cases

Emergency investment in healthcare	Announced short term spending on healthcare system, e.g., hospitals, masks, etc. Note: only record amount additional to previously announced spending	Record monetary value in USD. 0 - no new spending that day Blank - no data
Investment in vaccines	Announced public spending on Covid-19 vaccine development. Note: only record amount additional to previously announced spending	Record monetary value in USD. 0 - no new spending that day Blank - no data
Facial Coverings	Record policies on the use of facial coverings outside the home	0 - No policy 1 - Recommended 2 - Required in some specified shared or public spaces outside the home with other people present, or some situations when social distancing not possible 3 - Required in all shared or public spaces outside the home with other people present or all situations when social distancing not possible 4 - Required outside the home at all times regardless of location or presence of other people

Vaccination Policy	Record policies for vaccine delivery for different groups	0 - No availability 1 - Availability for ONE of following: key workers or clinically vulnerable groups (non elderly) or elderly groups 2 - Availability for TWO of following: key workers or clinically vulnerable groups (non elderly) or elderly groups 3 - Availability for ALL of following: key workers or clinically vulnerable groups (non elderly) or elderly groups 4 - Availability for all three plus partial additional availability (select broad groups or ages) 5 - Universal availability

Protection of elderly people	Record policies for protecting elderly people (as defined locally) in Long Term Care Facilities and/or the community and home setting	0 - no measures 1 - Recommended isolation, hygiene, and visitor restriction measures in LTCFs and/or elderly people to stay at home 2 - Narrow restrictions for isolation, hygiene in LTCFs, some limitations on external visitors and/or restrictions protecting elderly people at home 3 - Extensive restrictions for isolation and hygiene in LTCFs, all non-essential external visitors prohibited, and/or all elderly people required to stay at home and not leave the home with minimal exceptions, and receive no external visitors Blank - no data

Table 4: Climatic, environmental, clinical, health, Economic, pollution, social, and demographic factors

Covariates	Description	Reference
**Demographic**		
Total Population	Total number of the population	Nations 2019
Life expectancy	Life expectancy	Ritchie et al. 2020
Population density	Number of people divided by land area	Ritchie et al. 2020
Median age	Median age of the population	Ritchie et al. 2020
Rural population	Proportion of the population living in rural area	Bank 2020

Urbanization	Population in urban agglomeration of more than 1 million	Bank 2020
**Clinical**		
Cardiovascular diseases	Death rate due to cardiovascular diseases, per 100,000 people	Ritchie et al. 2020
Diabetes	Diabetes prevalence	Bank 2020
TB	Incidence rate of TB, per 100,000 people	Bank 2020
BCG	BCG vaccination coverage	Bank 2020
LRI	Lower respiratory infections rate, per 100,000 population	GHDx 2019
HIV	Prevalence of HIV	Bank 2020
Malaria	Reported cases of malaria	Bank 2020
BMI	Mean body mass index of 18+ years, in Kg/m2	WHO 2020
Raised BP	RRaised blood pressure (SBP≥140 or DBP≥90), age-standardized estimate	WHO 2020
Raised cholesterol	Raised total cholesterol (≥ 5.0 mmol/L), age-standardized estimate	WHO 2020
Communicable diseases	Communicable diseases and maternal, prenatal and nutrition conditions (include infectious and parasitic diseases, respiratory infections), per 100,000 populations	GHDx 2019
Cancer	Cancer prevalence	Ritchie et al. 2020
**Health**		
Testing capacity	Total tests for COVID-19 per 1,000 per 1 million population	Ritchie et al. 2020

Nurses	Number of nurses and midwifes per 1,000 population	Bank 2020
Physician	Number of physicians per 1,000 population	Bank 2020
GHS index	Global health security detection index	Initiative and the Johns Hopkins Center for Health Security 2019
**Economic**		
GDP	Gross domestic product per capita	Ritchie et al. 2020
GINI Index	GINI index (income inequality, 100= high)	Ritchie et al. 2020
Doing business	Ease of doing business rank 2019 (1=most business-friendly regulations)	Bank 2020
Human development index	Human development index	Ritchie et al. 2020
**Climatic**		
Temperature	Temperature in degree Celsius	Bank 2020
Rainfall	Rainfall in mm	Bank 2020
**Pollution**		
Pollution	PM2.5 air pollution, mean annual exposure (micrograms per cubic meter)	Bank 2020
UV radiation	Ultraviolet radiation exposure	Bank 2020
**Social**		
Internet filtering	Government internet filtering in practice (4= low)	Society 2021
Use of social media	Average people's use of social media to organize offline action (4= high)	Society 2021

Table 5: Estimated basic reproduction number and effective reproduction number (second wave) across countries

Country	R0	RE	Estimated RE
Afghanistan	1.59	1.22	1.71
Algeria	1.22	1.57	1.42
Armenia	1.44	1.77	1.72
Australia	5.19	1.36	4.35
Austria	3.66	1.41	3.26
Azerbaijan	1.31	1.38	1.57
Bahrain	1.32	1.14	1.47
Bangladesh	1.39	1.07	1.46
Belarus	1.71	1.21	1.79
Belgium	2.41	1.63	2.34
Brazil	1.28	1.36	1.41
Canada	1.88	1.18	2.06
Colombia	1.29	1.08	1.35
Cuba	1.64	1.1	1.81
Denmark	1.45	1.21	1.78
Dominican Republic	1.14	1.13	1.25
Egypt	1.43	1.15	1.45
Finland	1.36	1.15	1.72
France	1.64	1.24	2.03
Germany	3.06	1.51	2.93
Iran	1.48	1.11	1.46
Ireland	1.66	1.34	1.95
Israel	6.93	1.79	5.25
Italy	2.81	1.82	2.83
Japan	2.24	1.62	2.6
Kenya	1.3	1.54	1.39
Kyrgyzstan	2.45	1.68	2.09
Luxembourg	1.9	1.94	1.98
Malaysia	1.85	1.39	1.77
Mauritania	1.55	1.81	1.5
Mexico	1.23	3.08	1.35
Morocco	1.41	1.12	1.41
Namibia	1.28	1.39	1.41
Nepal	1.77	1.2	1.69
Netherlands	2.17	1.45	2.26
Norway	1.4	1.24	1.76
Oman	1.34	1.5	1.39
Pakistan	1.28	1.21	1.45

Panama	1.61	1.37	1.55
Peru	1.36	1.53	1.51
Portugal	3.85	1.36	3.25
Russia	1.74	1.2	1.82
Senegal	1.13	1.18	1.36
Singapore	2.82	1.73	2.92
South Africa	1.37	1.08	1.43
Spain	3.38	1.15	3.32
Sudan	1.8	2.31	1.66
Sweden	1.58	1.56	1.76
Switzerland	4.28	2.16	3.85
Turkey	2.52	2.82	2.13
United Arab Emirates	1.31	1.23	1.71
United Kingdom	2.01	1.43	2.15
United States	2.98	1.37	2.7
Uzbekistan	1.78	1.34	1.77
Zimbabwe	2.22	1.33	1.87

Bibliography

Bank, W. (2020), 'The World Bank DataBank', https://tinyurl.com/jp9bdd8. Accessed: 05-16-2021.

GHDx (2019), 'Global Health Data Exchange', http://ghdx.healthdata.org. Accessed: 05-16-2021.

Initiative, T. N. T. and the Johns Hopkins Center for Health Security (2019), 'The Global Health Security (GHS) Index', https://www.ghsindex.org/about/. Accessed: 05-16-2021.

Nations, U. (2019), 'World Population Prospects 2019', https://tinyurl.com/f2487w6w. Accessed: 05-16-2021.

Ritchie, H., Ortiz-Ospina, E., Beltekian, D., Mathieu, E., Hasell, J., Macdonald, B., Giattino, C., Appel, C., Rodés-Guirao, L. and Roser, M. (2020), 'Coronavirus Pandemic (COVID-19)', *Our World in Data* . https://ourworldindata.org/coronavirus.

Society, D. (2021), 'Digital Society Project', http://digitalsocietyproject.org/data/. Accessed: 05-16-2021.

WHO (2020), 'The Global Health Observatory data', https://www.who.int/data. Accessed: 05-16-2021.
